# Yellow Oleander (*Thevetia peruviana*) Toxicity from a Misrepresented Dietary Supplement: A Case Report

**DOI:** 10.5811/cpcem.54063

**Published:** 2026-08-05

**Authors:** Samantha E. Long, Allison Grim, Salil K. Bhandari, Hashim Q. Zaidi

**Affiliations:** The University of Texas Health Science Center at Houston, Department of Emergency Medicine, Houston, Texas

**Keywords:** yellow oleander, Thevetia peruviana, digoxin-like toxicity, dietary supplement, cardiac glycoside

## Abstract

**Introduction:**

The “lucky nut” is the seed of the yellow oleander plant, often sold as a medicinal supplement in unregulated markets and known to contain cardiac glycosides, which may cause life-threatening bradycardias and Digoxin-like toxicity upon ingestion. Diagnosis is typically clinical, with treatment including Digoxin immune Fab with supportive intensive cardiac care.

**Case Report:**

A 70-year-old male presented to the emergency department after ingesting one-fourth of a Peruvian nut he found at a local market to reduce his nocturnal polyuria. The patient brought a part of the Peruvian nut with him, which was identified as a seed of the yellow oleander. His clinical presentation was consistent with a digoxin-like toxicity, requiring multiple vials of Digoxin immune Fab and admission to the cardiac intensive care unit.

**Conclusion:**

Yellow oleander can cause a cardiac glycoside-related cardiotoxicity similar to a digoxin-like toxicity. Digoxin-like toxicity should be considered in the bradycardic patient with a recent ingestion of a plant or seed. It is important to obtain a thorough history including medication reconciliation and supplement use. Despite well-documented dangers, yellow oleander continues to appear as an unregulated ingredient or contaminant in dietary supplements. This case highlights the severity of oleander toxicity, the challenge of managing digoxin-like cardiac glycoside poisoning, and the public health risk posed by unintentional ingestion of supplements containing yellow oleander.

## INTRODUCTION

Yellow oleander is derived from a shrub with bright yellow-orange flowers found in tropical areas around the world. Its plant species include *Thevetia peruviana*, *Thevetia thevetioides*, and *Thevetia ovata*. These plants belong to the Apocynaceae botanical family with species long known to cause significant neurologic, gastrointestinal, and cardiac toxicity. Despite its known toxicity profile, it has been used in traditional and cultural medical practices for hemorrhoids, as an antiseptic and, most recently, marketed as a weight loss supplement sold through online retailers.^1^ This case report illustrates an accidental toxic ingestion of the seed of the yellow oleander, which was sold at a local market as a dietary supplement with purported health benefits against prostate enlargement. This product and similar dietary supplements are unregulated by the U.S. Food and Drug Administration (FDA). We present a case of accidental poisoning from the seed of the yellow oleander plant, known colloquially as “lucky nut,” to highlight clinical management of this toxidrome as well as the need for further consumer protection.

## CASE REPORT

**Image 1:**
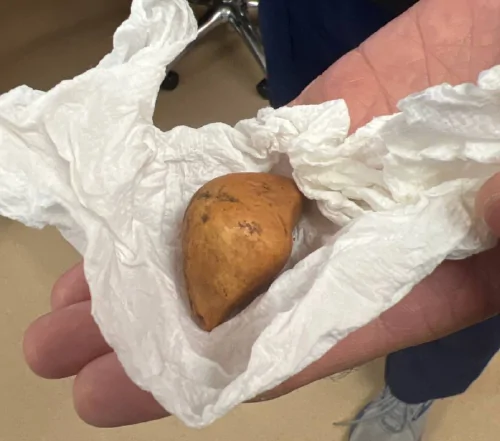
A specimen of the “lucky nut,” the seed of the yellow oleander (*Cascabela thevetia*) plant. The outer shell of this seed is characteristically hard and dark brown to black. It is highly toxic due to its concentration of cardiac glycosides, which can cause life-threatening bradycardia and cardiotoxicity in humans.

A 70-year-old male with a past medical history pertinent for hyperlipidemia, hypertension, and benign prostatic hyperplasia presented to the emergency department (ED) for nausea and vomiting. Thirty minutes prior to arrival, the patient reportedly ingested part of a seed of what he thought was a “Peruvian nut,” which he purchased at a local market. He was advised by a friend that this would help reduce his overnight urinary frequency caused by an enlarged prostate. The product he consumed was actually the seed of the yellow oleander plant, *Thevetia peruviana*, also known as the “lucky nut.” The patient brought the partially consumed nut with him to the ED, which allowed physicians to identify it using the Google search engine (Image 1). Given concerns for digoxin-like cardiac glycoside toxicity, poison control was contacted for assistance in management.

**Image 2:**
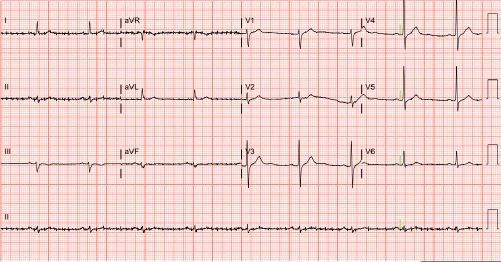
An electrocardiogram on initial presentation demonstrating sinus bradycardia in a patient whose presentation was consistent with a digoxin-like toxicity, requiring multiple vials of digoxin immune Fab and admission to the cardiac intensive care unit.

On presentation, the patient’s vitals were as follows: blood pressure, 132/74 millimeters of mercury; heart rate, 44 beats per minute; respiratory rate, 18 breaths per minute; and an oxygen saturation of 98% on room air. Electrocardiogram demonstrated sinus bradycardia with a rate in the 40s, with subjective “dizziness” concerning for cardiotoxicity (Image 2). The remainder of the physical exam showed normal capillary refill, warm extremities, clear breath sounds to auscultation, and a normal mental status and neurologic exam. Labs were fairly unremarkable, and despite concern for acute digoxin-like toxicity, initial serum potassium was normal at 4.5 millimoles per liter.

To manage the patient’s persistent bradycardia and concern for oleander-related cardiotoxic effects, consult from Poison Control recommended administration of Digoxin immune Fab to alleviate bradycardia. Initial dosing recommendations were to give five vials every 10 minutes with a maximum of 15 for a heart rate < 60. However, the facility had a limited supply and only four vials were stocked, all of which were administered to the patient. This did not improve the heart rate. Cardiac pacing was considered; however, because the patient showed no signs of hypoperfusion, it was deferred, but he was placed on the cardiac monitor and pads under close observation. The patient was transferred for higher level of cardiac care as he required ongoing Digoxin immune Fab and supportive cardiac intensive care with ongoing refractory bradycardia and signs of cardiotoxicity.

*CPC-EM Capsule*

What do we already know about this clinical entity?

*Yellow oleander contains potent cardiac glycosides that cause digoxin-like bradyarrhythmias and life-threatening cardiotoxicity.*

What makes this presentation of disease reportable?

*A toxic oleander seed sold as an unregulated dietary supplement caused severe bradycardia requiring extensive antidote therapy.*

What is the major learning point?

*Oleander ingestion can mimic digoxin toxicity and may require large, repeated doses of Digoxin immune Fab with close cardiac monitoring.*

How might this improve emergency medicine practice?

*This case raises awareness of supplement-related plant poisonings and reinforces early recognition and treatment of cardiac glycoside toxicity.*

Following transfer, the patient continued to have sinus bradycardia to the 30s and frequent sinus pauses. A digoxin level was obtained shortly following transfer was 4.04 nanograms per milliliter (ng/mL) (reference range: 0.5–2 ng/mL). Due to an elevated digoxin level and persistent bradycardia, additional doses of Digoxin immune Fab were given for a cumulative total of eight vials intermittently over 12 hours. Atropine 0.5 mg intravenous was also administered. Shortly after atropine administration, the patient’s heart rhythm converted to nonsustained atrial fibrillation with a rapid ventricular rate in the 140s. His mean arterial pressure ranged from 74–95 during that time, and atrial fibrillation quickly resolved without intervention.

The following day, the patient received an additional seven vials of Digoxin immune Fab for continued bradydysthymias, which were outsourced from surrounding hospitals. In total, the patient received 15 vials of Digoxin immune Fab during his admission. A repeat digoxin level improved to 0.99 ng/mL. He was observed for two more days in the hospital with brief episodes of self-limited sinus bradycardia without any further instability. The patient was ultimately discharged to home in stable condition in normal sinus rhythm and rate with significant symptomatic improvement.

## DISCUSSION

“Lucky nut” is the popular name of the seed of the yellow oleander plant (*Cascabela thevetia*), a shrub with bright yellow-orange flowers native to various tropical areas around the world, especially in Mexico and Central America. The misleading name, “lucky nut,” comes from its traditional use in some cultures as a talisman or charm, particularly in the West Indies.^1^ Diagnosis is typically clinical based on a history of ingestion and clinical findings. Similar to many cardiac glycosides, yellow oleander toxicity may exhibit digoxin-like effects including bradyarrhythmias, hypotension, and hyperkalemia. Clinicians should be aware of key initial management including early recognition of cardiac glycoside or digoxin-like toxicity, dysrhythmia management including initial cardiac stabilization, antidote therapy with Digoxin immune Fab, and the need for ongoing cardiac critical care.

Early poison control and cardiology consultations can aid in management and disposition. Activated charcoal may be considered for gastric decontamination if ingestion is less than 4 hours prior to evaluation. Cardiac glycosides are known to undergo enterohepatic recirculation. Therefore, multidose activated charcoal may be useful in reducing cardiotoxic effects, especially if Digoxin immune Fab is unavailable.^2^ There is no role for hemodialysis because cardiac glycosides have high protein binding and large volumes of distribution, making them poorly dialyzable.^3^

Digoxin immunoassays frequently cross-react with cardiac glycosides that bear similar structural moieties. Consequently, digoxin levels may be elevated such as in this case. However, this should be interpreted qualitatively rather than quantitatively to explain the presence of digoxin-like toxicity. Serum potassium level may correlate to the degree of sodium-potassium pump inhibition, with hyperkalemia > 5.5 milliequivalents per liter in adults being a potential treatment threshold for Digoxin immune Fab in suspected cardiac glycoside toxicity cases. Dosing of digoxin immune Fab is empiric based on cardiotoxicity signs. Initial empiric dosing of two to four vials is recommended for mild bradycardia or hypotension with further doses based on hemodynamic status and clinical severity.^3^

Digoxin immune Fab may be limited in effectiveness depending on the cardiac glycoside encountered given its specificity for digoxin. Supply and cost of the immune Fab may also restrict immediate availability. In addition to Digoxin immune Fab, bradyarrhythmias as a result of oleander toxicity can be mitigated with atropine, isoproterenol, or cardiac pacing in severe cases. However, use of atropine or other beta-adrenergic agonists such as isoproterenol increases the risks of tachydysrhythmias as seen in this case. If tachydysrhythmias are encountered, lidocaine may be an effective antiarrhythmic.^2^

This is an interesting case of accidental toxic ingestion that highlights the need for consumer education about supplements that are unregulated by the FDA. In September 2023, the U.S. Centers for Disease Control launched an investigation of over-the-counter health supplements thought to be contaminated with yellow oleander. As of 2024, the FDA has found that as many as 17 over-the-counter supplements contain yellow oleander despite not being listed as an active ingredient. The FDA determined that certain products labeled as tejocote (*Crataegus mexicana*) root or Brazil seed were adulterated after they were tested and found to be substituted with yellow oleander.

Despite the FDA contacting these sellers, recalled products can still be found on company websites or third-party merchants online such as eBay and Amazon.^4^ In fact, one study found that 66.7% of recalled supplements were still available for purchase at least six months after FDA recalls and remained adulterated with banned ingredients.^5^ It is critical for consumers to understand that the FDA does not approve manufacturers’ individual dietary supplements before they can be sold, and thus there is a risk of toxicity depending on the ingredients and concentrations present. It is prudent for clinicians to perform a diligent medication reconciliation on all patients with signs of cardiotoxicity and include any potential supplement use.

## CONCLUSION

Yellow oleander ingestion can cause severe, potentially fatal digoxin-like toxicity. Clinicians should suspect yellow oleander toxicity or other cardiac glycoside exposure when patients present with unexplained cardiotoxicity in the setting of dietary supplement ingestion of at-risk products. Enhanced regulation and increased public awareness are critical to reducing the incidence of plant-derived poisoning.
